# Notes and News

**Published:** 1896-10-31

**Authors:** 


### Notes and News.

(Collected and Communicated.)

The total collection of the Metropolitan Hospital Saturday Fund for the present year is £12,188, a substantial increase over that of 1895.

The moment has arrived when St. Mary's and the Southern Hospitals at Manchester must agree to accept the munificent offer of the David Lewis trustees, with the conditions attached to the gift, or lose for the poor of Manchester the benefits to be derived from it. It is stated that the objections have been raised principally by St. Mary's Hospital. The conditions are that the Southern and St. Mary's Hospitals should be merged into one, rebuilt on a new site in Stanley Grove, and coupled in some way with the name of David Lewis. Further, a few beds are to be reserved for educational purposes for Owens College Medical School. The offered gift amounted to £70,000, and the trustees now ask for a final acceptance or refusal.

We are informed that when the Plaistow Fever Hospital was opened some months ago application was made to the West Ham Public Libraries Committee for a supply of books for the staff and patients. The chief librarian, Mr. Cotgreave, interested himself in the matter, and finally formulated a scheme whereby a special branch of the West Ham Public Libraries has been instituted within the hospital, a small room being fitted up for the purpose, the library presenting spare or duplicate volumes to the hospital. The matron acts as honorary librarian. This arrangement provides not only a permanent and increasing collection of books, but also many magazines and papers. So far books are only issued to the staff, but if the great difficulty of disinfection can be overcome, convalescent patients, on application through the sister of their wards, will also be supplied. Our correspondent remarks that the institution of a hospital branch of a public library is probably a new departure, and one which might be adopted in other municipal hospitals with advantage.

Dr. George Duffy has been elected President of the Royal College of Physicians in Ireland.

A new out-patients' wing and laundry has been opened at the Loughborough Hospital by Lady Alice Packe. The cost is estimated at £579.

The sum of one thousand guineas has been promised by friends of the Great Northern Hospital to assist in clearing off the debt due to the bankers, providing the remainder of the money necessary is forthcoming by the end of the year.

A bazaar was held this week in aid of the building fund of the Kettering and District General Hospital. The takings of the first day amounted to £1,200. The neighbourhood of Kettering is a wealthy one, so we hope that the deficiency of £2,500 which exists in the funds will be nearly swept away.

The New Pathological Institute at Glasgow was opened recently. The new buildings will be of great value, not only to the Western Infirmary, with which they are actually connected, but also to the University Medical School. The opening address was delivered by Professor Gairdner. The institute cost upwards of £15,000.

It will be good news for London and provincial hospitals that Mr. Bancroft is about to give a series of readings on their behalf should the first prove a success, of which there can be but little doubt. The pioneer reading will be given on the evening of November 23rd, in the large Queen's Hall, Langham Place, and the subject Mr. Bancroft has chosen is Charles Dickens's "Christmas Carol."

The foundation-stone of the new "David Lewis" Northern Hospital at Liverpool was laid last week by the Countess of Derby as Lady Mayoress of the city. The hospital, which originated in 1833 with thirty-three beds, will now be arranged to accommodate 200 patients. The David Lewis trustees will bear the whole cost of the new buildings, which is likely to exceed £80,000, the Corporation contributing part of the site and £10,000.

Last week a Trades and Arts Exhibition was opened at Middlesbrough in aid of local medical charities. A similar exhibition was held last year for the same purpose, when 25 per cent. of the net profits were handed over to the charities. This year, owing to the generosity of Councillor Phillips, the mayor, who has guaranteed the committee against loss, 45 per cent. of the net profits is to go to two of the institutions selected to be benefited, and 10 per cent. to the third. The exhibition, which is excellently arranged, opened with every prospect of a brilliant success.

The children's ward at the Infirmary, Barnstaple, was opened by the patron of the institution, Earl Fortescue, last week. The ward now devoted to the use of children is not a new one, but is a previously used ward adapted to its present purpose. The accommodation is for twelve patients, the cots have all been given, and the expense to which the infirmary has been put is only for the structural alterations and fittings. Major Hogg started the movement of presenting the cots by giving two himself. The ward is to be named the "Victoria."

